# Preparation of Poly(acrylic acid-acrylamide/starch) Composite and Its Adsorption Properties for Mercury (II)

**DOI:** 10.3390/ma14123277

**Published:** 2021-06-14

**Authors:** Wenjuan Zhu, Zhiyong Yang, Akram Yasin, Yanxia Liu, Letao Zhang

**Affiliations:** 1 School of Chemical and Environmental Engineering, Xinjiang University of Engineering, Urumqi 830026, China; yzy11518602@163.com (Z.Y.); zhanglt@ms.xjb.ac.cn (L.Z.); 2 Xinjiang Technical Institute of Physics and Chemistry, Chinese Academy of Sciences, Urumqi 830011, China; akram@ms.xjb.ac.cn (A.Y.); liuyanxia@ms.xjb.ac.cn (Y.L.)

**Keywords:** composite, starch, acrylic acid, acrylamide, adsorption

## Abstract

The poly(acrylic acid-acrylamide/starch) composite was synthesized by solution polymerization, aiming to adsorb mercury (II) in water. The resulted copolymer was characterized by particle size exclusion chromatography (SEC), Fourier transform infrared spectroscopy (FTIR), thermogravimetry (TG), scanning electron microscopy (SEM) and dynamic light scattering particle size analyzer (DLS). It turned out that starch was successfully incorporated with the macromolecular polymer matrix and played a key role for improving the performance of the composites. These characterization results showed that the graft copolymer exhibited narrow molecular weight distribution, rough but uniform morphology, good thermal stability and narrow particle size distribution. The graft copolymer was used to remove Hg(II) ions from aqueous solution. The effects of contact time, pH value, initial mercury (II) concentration and temperature on the adsorption capacity of Hg(II) ions were researched. It was found that after 120 min of interaction, poly(acrylic acid-acrylamide/starch) composite achieved the maximum adsorption capacity of 19.23 mg·g^−1^ to Hg(II) ions with initial concentration of 15 mg·L^−1^, pH of 5.5 at 45 °C. Compared with other studies with the same purpose, the composites synthesized in this study present high adsorption properties for Hg(II) ion in dilute solution. The adsorption kinetics of Hg(II) on the poly(acrylic acid-acrylamide/starch) composite fits well with the pseudo second order model.

## 1. Introduction

Starch is a kind of natural and renewable macromolecule carbohydrate, which has the characteristics of broad obtaining source, low cost, good biodegradability and no secondary pollution [1,2]. Therefore, modified starch adsorbent has become a research hotspot in removing pollutants from water [3,4,5]. Starch does not have good water resistance and processability. It can be graft-copolymerized with vinyl carboxylic acid monomer to make composite materials to enhance its swelling, water resistance and adsorption properties, expanding the application of starch in the functional material field. Starch based copolymer adsorbents have been studied for many years, but most of the composite materials with high adsorption performance are aiming at high concentration of metal ion solution [6,7]. In this study, a starch-based adsorbent with superior adsorption capacity for low concentration mercury (II) ions was synthesized. The material exhibits narrow molecular weight distribution, good thermal stability and uniform surface morphology.

In recent decades, heavy metal pollution in water environment has attracted widespread attention, especially mercury ion pollution, which is the most toxic [8]. Due to the characteristics of persistence and bioaccumulation, the ingestion of mercury ions will cause serious damage to human body and organisms [9,10]. Hg(II) ions can form Hg-S bonds with cysteine of protein chain, which can cause damage to central nervous system, cardiovascular system, kidney and bone [11]. Therefore, it is of great significance to remove mercury (II) ions in aqueous solution for the protection of ecological environment and human health [12,13]. Mercury (II) ions in the environment mainly come from chlor alkali, battery manufacturing, chemical fertilizer, paper making, plastics, refining and paint industries [14,15]. The methods of mercury removal in water include chemical precipitation, coagulation, ion exchange, membrane filtration and adsorption [16,17]. Since most adsorption processes are exothermic and spontaneous. Adsorption is the most common method for removing mercury ions from water. Although the adsorption capacity of activated carbon is very strong, high production cost and poor selectivity limit its extensive usage [18,19]. As a result, the adsorbent composite that can remove Hg(II) ions in water have attracted great interest [20,21]. In practice, there are not many cases of high concentration mercury ion contamination. According to the comprehensive sewage discharge standard GB8978-2005 in China, the concentration of mercury ions in the discharged sewage shall not exceed 0.05 mg·L^−1^ [22]. In this study, a composite material for treating lower concentrations of mercury ions was synthesized in order to further investigate the ability of the material to deal with actual mercury ion wastewater.

## 2. Materials and Methods

### 2.1. Materials

Acrylic acid (AA) was distilled under reduced pressure to eliminate the inhibitor. Acrylamide (AM) was purified by recrystallization from benzene. Potassium persulfate (KPS) was recrystallized from water. N, N-methylenebisacrylamide (MBA), sodium hydroxide (NaOH), mercuric chloride (HgCl_2_) and methanol were of analytical grade and used as received. These chemicals were supplied by Tianjin Chemical Reagent Factory, Tianjin, China. Starch was purchased from JinHui Corn Products, Binzhou, Shandong, China.

### 2.2. Synthesis of PAA-AM/St Superabsorbent Composite

PAA-AM/St composite was prepared as follows: For a typical experiment, 12 mL AA was neutralized with 8M sodium hydroxide solution in an ice bath under stirring. Then this neutralized AA and 10.0 g AM were added to 80 mL distilled water in 250 mL four-neck flask assembled with a reflux condenser, a thermometer and a N_2_ inlet tube. Then 0.15 g of MBA was added, the mixture was magnetically stirred for 30 min before 2.0 g of starch were added. After the starch was evenly dispersed, the mixture was heated to 70 °C gradually while 0.12 g of KPS was added. The polymerization reaction was continued for additional 30 min at 70 °C. The whole process was under the protection of nitrogen atmosphere. The resulting granular product was placed in Soxhlet extractor, washed and purified with the mixture of methanol and water (6:1 by volume), dried at 65 °C for 48 h in a vacuum oven. All samples were milled and screened into particles with size of 40–80 mesh for further tests. The PAA-AM/St composite was obtained.

### 2.3. Characterization

The molecular weight and the molecular weight distribution analysis of PAA-AM/St were carried out by Waters e2695 size exclusion chromatography (SEC) (Water Pvt. Ltd., Milford, MA, USA), equipped with waters 2414 differential detector and 6.0 mm × 150 mm HSPgel^TM^ column [23]. The flow rate was fixed at 0.5 mL min^−1^ under 30 °C. Dextran and sodium polystyrene sulfonate were used as standard 3450 polymers [24]. The FTIR spectra of raw starch and PAA-AM/starch composite were measured by a DIGILAB FTS 3000 FT-IR spectrophotometer (Digilab Inc., Boston, MA, USA) under the transmission mode. The ground samples were dispersed into KBr pellets at mass ratio of 1:60. At spectral resolution of 2 cm^−1^, the data were collected in the wavenumber range of 4000–400 cm^−1^ after 64 scans. SEM studies were carried out on a JSM-5600 LV scanning electron microscope (Japan Electron Optics Laboratory Co. Ltd., Tokyo, Japan) after coating the sample with platinum film using an acceleration voltage of 20 kV. Thermogravimetric analysis (TGA) was carried out in a Perkin-Elmer TGA-7 thermogravimetric analyzer PE TG/DTA 6300 instrument (Perkin-Elmer Inc., Boston, MA, USA) over a temperature range of 20–800 °C, at a nitrogen flow rate of 50 mL min^−1^ under a heating rate of 10 °C min^−1^. The particle size distribution of the composite was measured by dynamic light scattering (DLS) on a NICOMP 380N3000 nanoparticle size analyzer (PSS, Santa Barbara, CA, USA). The laser wavelength was 514.5 nm and the solvent was water.

### 2.4. Adsorption of Hg(II) Ions

A total of 50 mL of Hg(II) solution with an initial concentration of 15 mg·L^−1^ was added into a conical flask. The pH of the solution was adjusted to 5.5 with hydrochloric acid of pH = 2 and NaOH solution of pH = 12. Then 0.01 g of graft copolymer was put into the solution for Hg(II) ion adsorption experiment. The adsorption equilibrium was achieved by stirring at 120 rpm for 2 h at 45 °C. After centrifugation, the concentration of Hg(II) in the supernatant was determined by atomic absorption spectrophotometer. The adsorption capacity (mg·g^−1^) of the graft copolymer for Hg(II) ion was calculated by Formula (1):

(1) $$Q_{t}=\frac{\left(C_{i}-C_{t}\right)}{W}V$$

where *Q_t_* is the adsorption capacity of metal ions at time t. *C_i_* and *C_t_* are the initial and final concentrations of Hg(II) ion (mg·L^−1^), respectively. *V* is the volume of Hg(II) ion solution and *W* is the mass of graft copolymer.

### 2.5. Regeneration of the PAA-AM/St Composite

The PAA-AM/St composite that reached the saturated adsorption of Hg(II) ions was put into a 250 mL flask, in which 65 mL of 1 mol·L^−1^ NaOH solution was added. Heating the mixture to 55 °C for 1.5 h under refluxing, the desorption experiment was carried out. After the desorption, the mixture was centrifuged and the supernatant was discarded. The composite was precipitated again by adding the mixture of methanol and water (volume ratio 5:1). After being washed several times with a mixture of distilled water and methanol (1:1 by volume), the composite was then dried in a vacuum oven.

## 3. Results and Discussion

**Figure 1:**
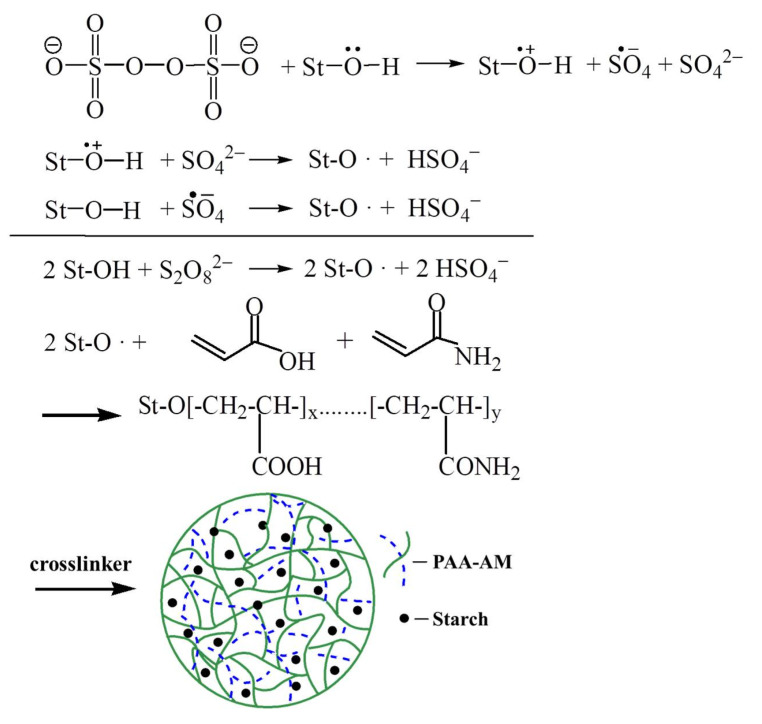
Schematic illustration of polymerization.

The starch-g-poly (acrylic acid acrylamide) graft copolymer was prepared by solution polymerization through free radical mechanism [24,25]. The graft copolymerization was carried out according to the stoichiometric equation (shown in Figure 1).

### 3.1. Measurement of Molecular Weight

**Figure 2:**
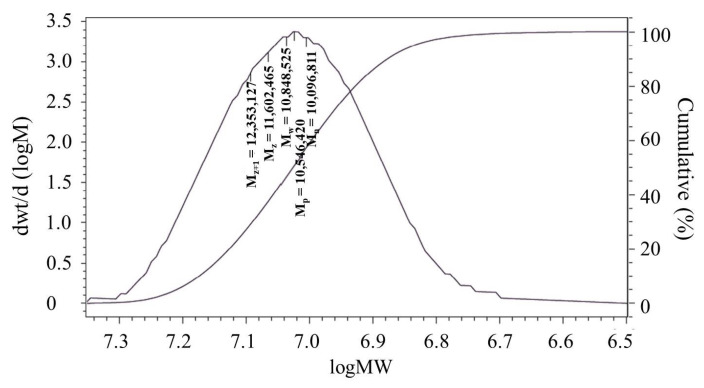
The molecular weight and the molecular weight distribution of PAA-AM/St.

The molecular weight of the PAA-AM/St composite was determined by SEC. The composite was run in the chromatographic column and the molecular weight distribution curve was constructed by fraction elution and calibration plot. M_n_, M_p_, M_w_, M_z_ and M_z+1_ values were located in Figure 2. The relative standard deviation (RSD) of the measurement results in Figure 2 were listed in Table 1. All the RSD values are less than 5%, which indicates that the SEC method performed is accurate and reliable [26]. PDI is defined as the ratio of M_w_ to M_n_ [27]. Generally speaking, the larger the PDI value is, the wider the molecular weight distribution is, the smaller the PDI value is, the narrower the molecular weight distribution is [28]. The PDI values of starch and PAA-AM/St composite calculated from Table 1 are 1.06 and 1.07, respectively. Both of them are less than 1.10, which means that the molecular weight distribution of the PAA-AM/St composite is relatively narrow, and the chain length of the polymer is relatively uniform [29].

### 3.2. FT-IR Spectra

**Figure 3:**
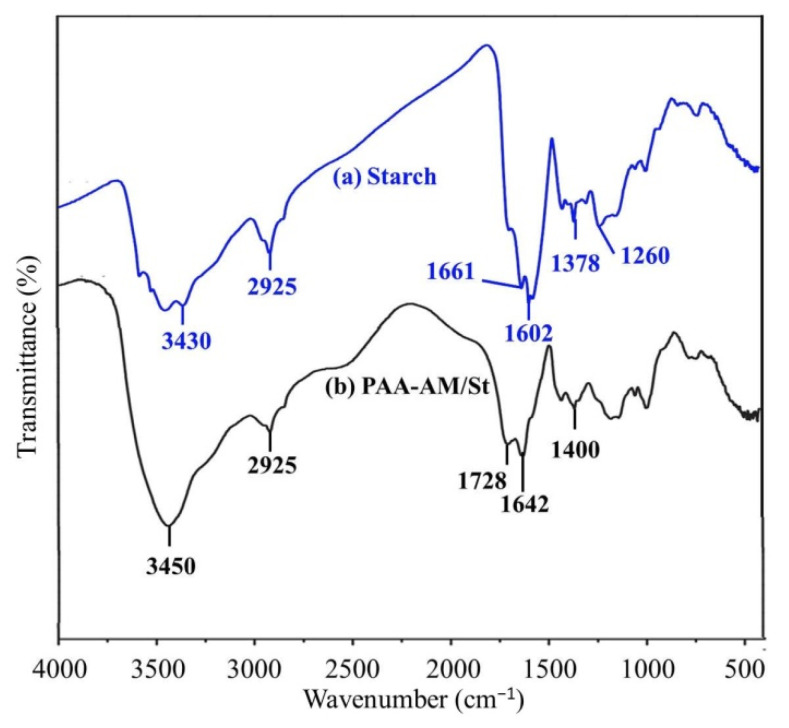
FT-IR spectra of (**a**) starch and (**b**) PAA-AM/St composite.

The infrared spectra of starch and graft copolymer are shown in Figure 3. The peak at 1642 cm^−1^ was the stretching vibration band of carbonyl group in AM and at 1728 cm^−1^ was caused by stretching vibration of C=O in AA (Figure 3b). There were no such peaks in starch (Figure 3a). The broad and strong absorption band near 3450 cm^−1^ represents the stretching vibration of -OH group in AA and -NH group in AM [30]. The 1400 cm^−1^ is related to the stretching vibration of C-N bond in graft copolymer [31]. Since the homopolymer was completely removed, the peaks at 1642 cm^−1^ and 1728 cm^−1^ indicate that the poly (acrylic acid-acrylamide) chain was successfully grafted into the starch structure.

### 3.3. Thermal Stability

**Figure 4:**
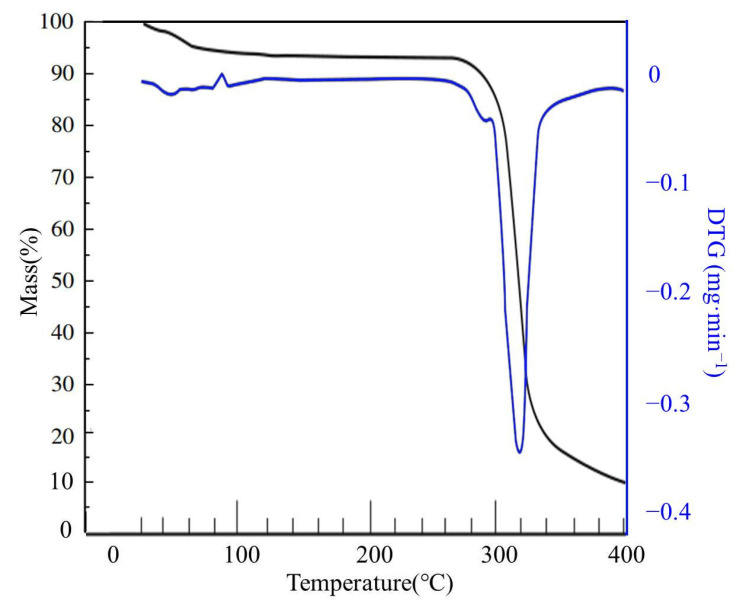
The TG and DTG curves of starch.

**Figure 5:**
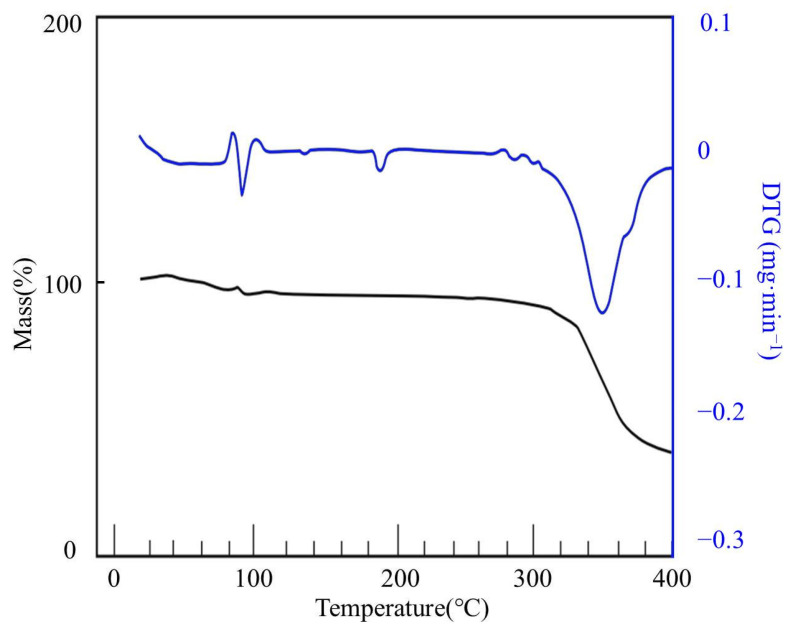
The TG and DTG curves of PAA-AM.

**Figure 6:**
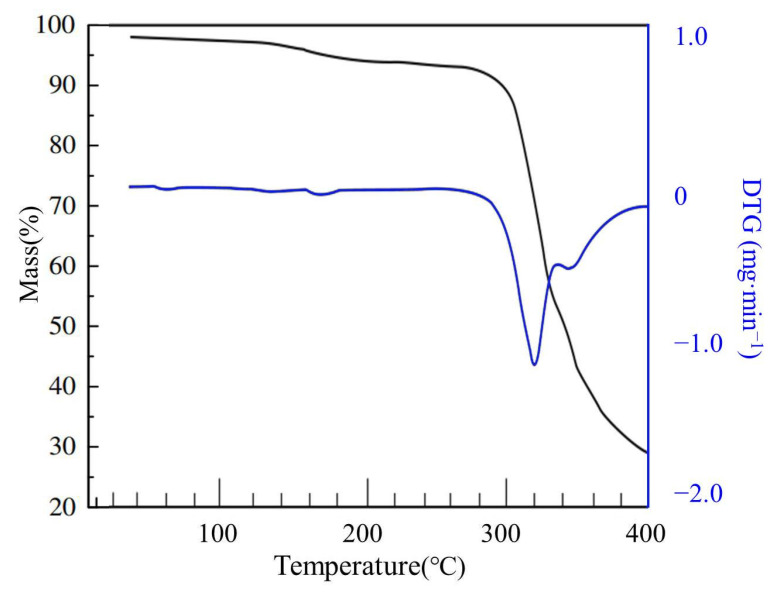
The TG and DTG curves of PAA-AM/St.

The thermogravimetric curve of starch is shown in Figure 4. It can be seen that the starch had undergone three stages of decomposition, the main mass loss occurred in the temperature range of 264–337 °C and the weight loss rate was up to 68% at 316 °C [26]. The thermogravimetric curve of the PAA-AM is shown in Figure 5. It can be seen that the PAA-AM also underwent three stages of decomposition, the main weight loss occurred between 300 and 368 °C. The maximum weight loss happened at 347 °C. There were two characteristic peaks between 263 and 331 °C and 331 and 400 °C of PAA-AM/St, and the significant weight loss occurred at 316 °C and 342 °C, respectively (as shown in Figure 6). The weight loss between 263 and 331 °C corresponded to the collapse of the main structure of starch, while the weight loss between 331 and 400 °C was due to the collapse of the chain structure of PAA-AM copolymers [27,28]. The results show that the poly (acrylic-acrylamide) chain was successfully grafted onto the starch structure. The total weight loss of starch and PAA-AM copolymer was 85% and 67% respectively at 400 °C, while that of PAA-AM/St composite was 73%. Therefore, it can be concluded that the grafting poly (acrylic acid-acrylamide) with starch is a promising way to obtain thermally stable composite than pure starch.

### 3.4. Morphology Analysis

**Figure 7:**
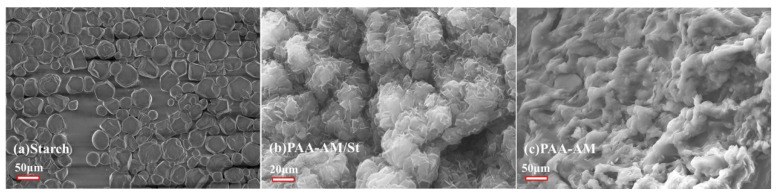
SEM images of (**a**) starch, (**b**) PAA-AM/St and (**c**) PAA-AM.

The surface morphology of the starch, PAA-AM and PAA-AM/St were observed using scanning electron microscopy (SEM). It can be seen from Figure 7a that the electron micrographs of pure starch samples clearly exhibited irregularly shaped granular structure. Figure 7b shows that integrating starch particles into PAA-AM copolymer can form composites with large specific surface area and uniform morphology. Evidently, incorporating starch granules onto PAA-AM matrix generated a flower shaped structure with starch evenly packaged by PAA-AM. With the formation of a large number of gaps and ravines on the surface, the specific surface area of the composite increased sharply, which makes it easier to carry more active adsorption sites. Therefore, Hg(II) ions can be easily adsorbed. The morphology of PAA-AM/St (Figure 7b) can be divided into three parts. The bright bulges are presumed to be the furrows in the composite, the gray area are probably the gullies between the furrows, and the dark regions are speculated to be cavities created during graft copolymerization [32]. Combining all these three parts, PAA-AM/St was composed of fine particles, possessing not only a uniform and stable structure but also very large surface area. Comparing with the morphology of PAA-AM shown in Figure 7c, it is easy to find out that the introduction of starch makes the surface of the PAA-AM/St composite much coarser than that of PAA-AM. Not any significant fracture between starch and PAA-AM matrix is observed in the composite, which proved that starch and PAA-AM chains were successfully integrated.

### 3.5. DLS Study

**Figure 8:**
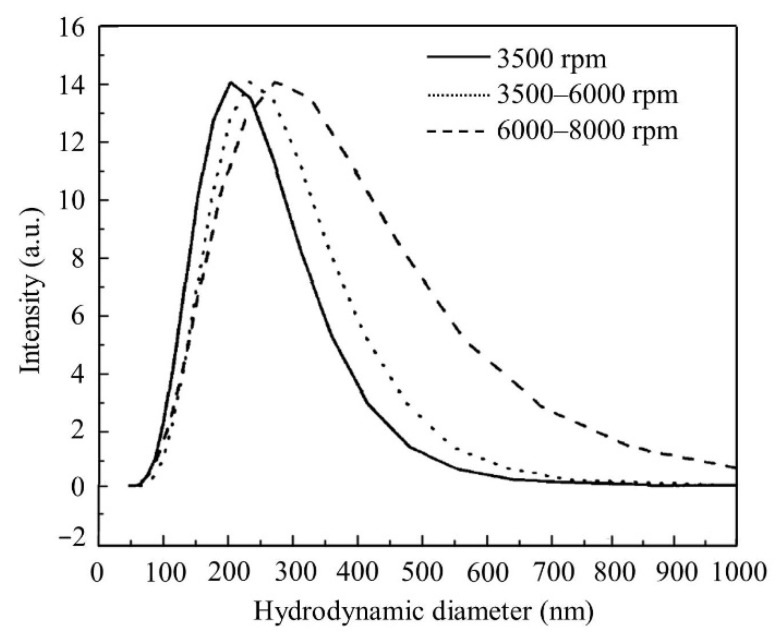
Particle size and distribution of the PAA-AM/St composite.

The particle size distribution of the composite was analyzed by dynamic light scattering test. The results are shown in Figure 8, from which it can be found that the particle diameter distribution of the composite was not significantly different under different centrifugal velocities. It can be seen from the figure that the most probable particle size of PAA-AM/St was 205 nm under 3500 rpm centrifugation, and the particle size distributed in the range of 50–520 nm. The maximum particle size of PAA-AM/St under 6000–8000 rpm centrifugation was 268 nm, and the particle size distribution was in the range of 50–600 nm. This means that even at high centrifugal strength, PAA-AM/St particles of different sizes will not be separated [33].

### 3.6. Optimization of Hg(II) Ions Adsorption Conditions

#### 3.6.1. Effect of Contact Time on the Adsorption of Hg(II) Ions by the Graft Polymer

**Figure 9:**
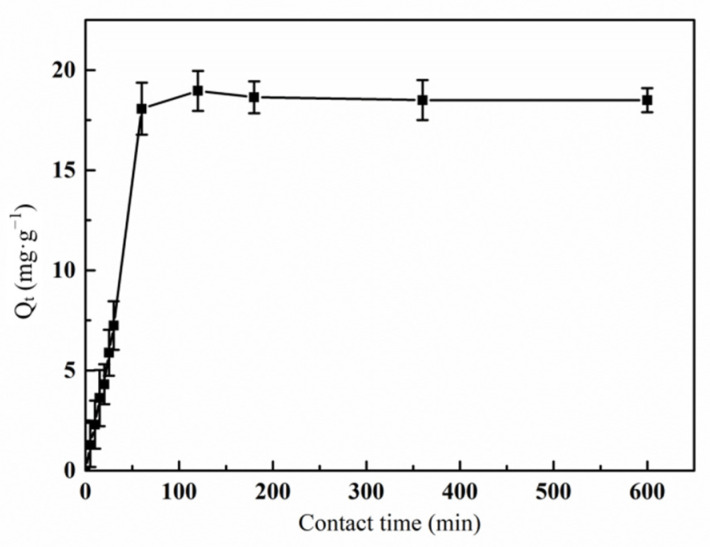
Effect of contact time on the adsorption of Hg(II) ions by graft polymer (pH value 5.5; initial concentration of Hg(II) ion 15 mg·L^−1^; treatment temperature 45 °C).

Firstly, the effect of contact time on the adsorption of Hg(II) ions was investigated. Figure 9 showed the relationship between the contact time and the adsorption capacity of PAA-AM/St composite. Within a certain time range (0–600 min), the adsorption of Hg(II) ions by graft copolymers increased sharply at first (0–50 min) and then tended to equilibrium. Maximum adsorption occurred at 120 min. This is because the surface area of the graft copolymer is very large, carrying a large number of adsorption active sites [34]. As the adsorption process proceeds, the vacant active sites are gradually occupied. When the adsorption active sites are gradually saturated with the Hg(II) ions, the adsorption process reaches equilibrium [35].

#### 3.6.2. Effect of pH Value on the Adsorption of Hg(II) Ions by the Graft Polymer

**Figure 10:**
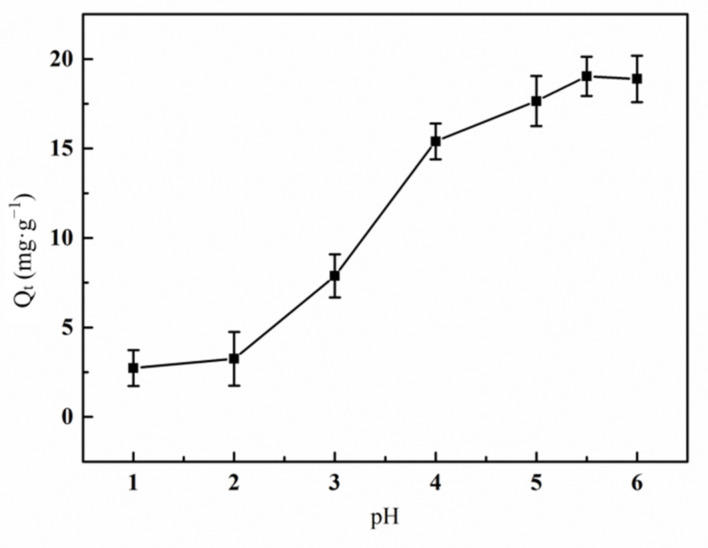
Effect of pH value on the adsorption of Hg(II) ions by graft polymer (contact time 120 min; initial concentration of Hg(II) ion 15 mg·L^−1^; treatment temperature 45 °C).

The change of adsorption capacity of graft copolymer for Hg(II) ion with pH value was shown in Figure 10. It can be seen from the figure that the pH of the solution had a significant effect on the adsorption of Hg(II) ions by the graft copolymer. In the low pH environment, H^+^ in the solution and the lone pair electrons on the N atom of the -NH_2_ group in AM formed hydrogen bonds, which makes the -NH_2_ group unable to combine with positively charged Hg(II) ions. Meanwhile, with the existence of massive H^+^, the carboxyl group from AA almost always kept the protonated form -COOH, which does not have the ability to combine with mercury (II) ions, either [36]. The adsorption capacity of the graft copolymer for Hg(II) increased with the raise of pH, and decreased after reaching a certain pH value (5.5). When the pH value was 5.5, the adsorption capacity reached the top. This is because while the pH value kept on increasing, -COOH dissociated into -COO^−^, which can combine with positively charged Hg(II) ions [37]. As the effective adsorption sites are gradually occupied, the adsorption process will slow down and gradually tend to equilibrium.

#### 3.6.3. Effect of Initial Hg(II) Ions Concentration on the Adsorption Property by Graft Polymer

**Figure 11:**
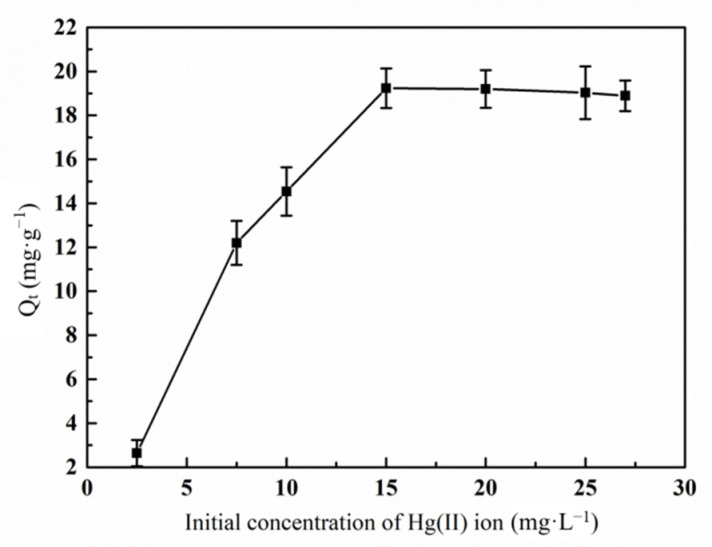
Effect of initial concentration of Hg(II) ion on the adsorption of Hg(II) ions by graft polymer (contact time 120 min; pH value 5.5; treatment temperature 45 °C).

The effect of the adsorption capacity of Hg(II) ions on the graft copolymer with the initial Hg(II) ions concentration is shown in Figure 11. Obviously, when the initial concentration of Hg(II) ion increased from 2.5 to 15.0 mg·L^−1^, the binding sites on the graft copolymer were sufficient, and the low concentration Hg(II) ion can be fully adsorbed on the active sites in an orderly manner [38]. With the increase of initial concentration of Hg(II), there were too many Hg(II) ions in the unit volume solution. Parts of Hg(II) ions quickly occupy the active sites on the surface, forming a positive layer on the surface of the graft copolymer, which blocks the further adsorption of Hg(II) ions. Therefore, the adsorption capacity decreased gradually.

The effect of time on Hg(II) adsorption capacity at different initial concentrations was investigated. The data is then fitted by the pseudo first order model (Formula (2)) and pseudo second order model (Formula (3)) to conduct adsorption kinetics study. Plot log(*q_e_* − *q_t_*) versus *t* to perform pseudo first order model fitting. Plot *t*/*q_t_* versus t to perform pseudo second order model fitting [24]. The relevant parameters are listed in Table 2. It can be seen from the R^2^ value that the adsorption kinetics of Hg(II) on the composite fits well with the pseudo second order model.

(2) $$\log(q_{e}-q_{t})=\log q_{e}-\frac{k_{1}}{2.303}\;t$$

(3) $$\frac{t}{q_{t}}=\frac{1}{k_{2}q_{e}^{2}}+\frac{t}{q_{e}}$$

In the above formula, *k*_1_ and *k*_2_ are the rate constants of the pseudo first-order model and the pseudo second-order model, respectively; *q_e_* and *q_t_* are the adsorption capacities at equilibrium and at time *t*, respectively.

#### 3.6.4. Effect of Temperature on the Adsorption of Hg(II) Ions by the Graft Polymer

**Figure 12:**
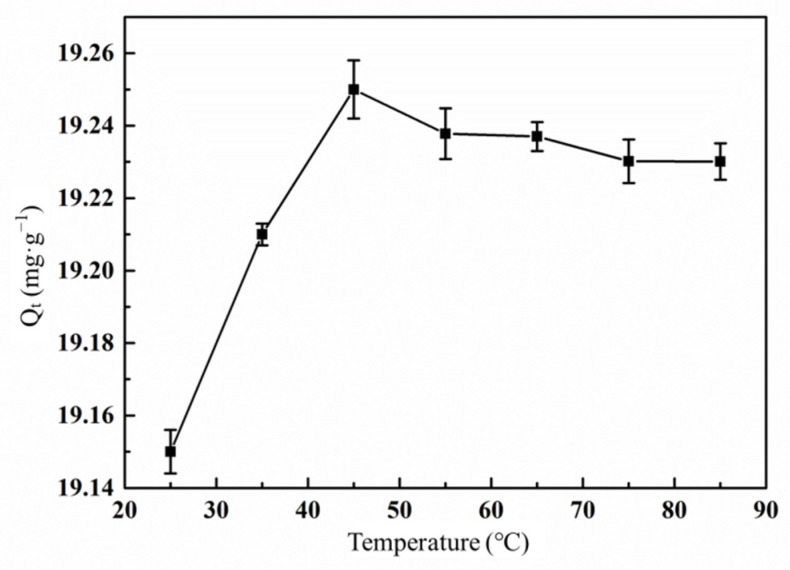
Effect of treatment temperature on the adsorption of Hg(II) ions by the graft polymer (contact time 120 min; pH value 5.5; initial concentration of Hg(II) ion 15 mg·L^−1^).

The influence of temperature on the adsorption capacity of metal ions mainly depends on the chemical structure of the adsorbent surface and the physicochemical state of the solution. When the temperature rises from 25 to 45 °C, the adsorbent swells well in the Hg(II) solution thanks to the thermal energy, exposing more active adsorption sites on the adsorbent surface. Therefore, in this temperature range, the increase of temperature is helpful to enhance the adsorption. However, the spontaneous adsorption reaction is an exothermic process. Generally speaking, in order to obtain a better adsorption effect, the treatment temperature should not be too high [39]. Figure 12 further confirms this view. When the solution temperature exceeds 45 °C, the increase of temperature cause the decrease of adsorption capacity [40].

### 3.7. Recyclability Study

**Figure 13:**
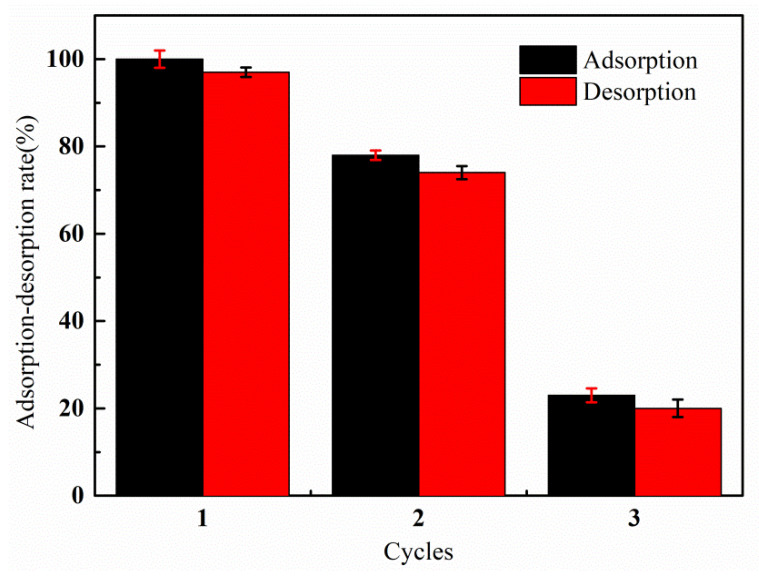
Adsorption–desorption rate in 1 mol·L^−1^ NaOH solution under different cycles.

The results of the adsorption–desorption experiments are shown in Figure 13. It can be seen that the adsorption capacity of the graft copolymer on the Hg(II) ion decreased to 78% of the original adsorption capacity after the treatment of the 1 mol·L^−1^ NaOH solution. After the third cycle, the adsorption capacity decreased to 23% of the original. The damage of the 1 mol·L^−1^ NaOH solution caused to the PAA-AM/St composite is catastrophic, but the composite can still reach 78% of the original adsorption capacity when it is recycled. This indicates that the PAA-AM/St composite prepared in this study can put up a good adsorption performance for Hg(II) ion under a severe situation.

### 3.8. Comparison Study

The adsorption capacities of various composite materials for the Hg(II) ion at different initial concentrations were summarized in Table 3. According to the data in the comparison table, when the initial concentration of Hg(II) ion varied between 100 and 4000 mg·L^−1^, all kinds of composites showed good adsorption capacity for Hg(II) ion (94.33–798.92 mg·g^−1^). When the initial concentration of Hg(II) ion was less than 100 mg·L^−1^, the adsorption capacities of the composites for Hg(II) ion ranged from 5.64 to 21.52 mg·g^−1^. Considering that the concentration of Hg(II) ion is generally not high in practical pollution, 15 mg·L^−1^ was chosen as the initial concentration of Hg(II) ion in this study, and it was found that the PAA-AM/St composite synthesized in this study achieved a satisfactory adsorption effect on the Hg(II) ion.

## 4. Conclusions

The starch-g-poly (acrylic acid-acrylamide) composite was synthesized from starch, acrylic acid and acrylamide. SEC characterization indicated that the graft copolymer exhibited narrow molecular weight distribution. The FT-IR spectra showed that starch was successfully integrated with the macromolecular polymer matrix and played a key role for improving the performance of the composites. The TG tests revealed that the thermal stability of PAA-AM/St was superior to that of PAA-AM and starch within 300 °C. The SEM images declared that the PAA-AM/St composite embodied rough but uniform morphological feature. The DLS analysis explained that the composite had a narrow particle size distribution and will not be separated even at high speed centrifugation conditions.

The graft copolymer was also used to remove heavy metal ions from aqueous solution, and its adsorption performance for mercury (II) in aqueous solution was studied. The influence of contact time, pH value, initial Hg(II) ions concentration and treatment temperature on the adsorption capacity were researched. The results indicated that the composite achieved the maximum adsorption of 19.23 mg·g^−1^ to Hg(II) ions in aqueous solution whose initial concentration of Hg(II) ion was 15 mg·L^−1^. Compared with the published literature, this adsorption capacity is a good result in the reported studies on the adsorption of Hg (II) ions by composite materials. The PAA-AM/St composite synthesized in this research shows excellent adsorption for Hg(II) ions in dilute solution, which is of great practical significance in the field of treating wastewater actually polluted by Hg (II) ions. The graft copolymer can be used as Hg(II) ion adsorbent to effectively remove toxic Hg(II) ions in wastewater. However, considering the fact that the concentration of Hg(II) ions in most wastewater is not high in practical application, more attempt should be made to improve the treatment efficiency of composite materials for wastewater with low concentration of heavy metal ions in further research.

## Figures and Tables

**Table 1:** Different average molecular weights of starch and PAA-AM/St.

Sample	M_n_	RSD	M_p_	RSD	M_w_	RSD	M_z_	RSD	M_z+1_	RSD
Starch	876,032	4.7%	906,360	4.0%	933,126	2.3%	936,921	0.9%	967,686	0.7%
PAA-AM/St	10,096,811	4.5%	10,546,420	3.0%	10,848,525	2.1%	11,602,465	1.0%	12,353,127	0.9%

**Table 2:** The relevant parameters for pseudo first order adsorption and pseudo second order adsorption.

Initial Concentration of Hg(II) (mg·L^−1^)	Pseudo First Order Model		Pseudo Second Order Model	
	k_1_ (min^−1^)	R^2^	k_2_ (g·mg^−1^·min^−1^)	R^2^
3.0	2.3 × 10^−2^	0.325	3.3 × 10^−3^	0.999
7.0	3.1 × 10^−3^	0.103	8.9 × 10^−4^	0.999
9.0	3.3 × 10^−3^	0.386	8.1 × 10^−4^	0.999
15.0	4.1 × 10^−3^	0.468	6.4 × 10^−4^	0.999
26.5	4.3 × 10^−3^	0.369	5.2 × 10^−4^	0.999
30	4.2 × 10^−3^	0.412	4.9 × 10^−4^	0.999

**Table 3:** Adsorption capacity of various composite materials for Hg(II) ion at different initial concentrations.

Composites Used as Adsorbent	Initial Concentration of Hg(II) ion (mg·L^−1^)	Q_t_ (mg·g^−1^)	Reference
PAN-AA/AMP	200	221.23	[1]
PAN-AA	200	202.43	[1]
St-PEG-PAA	300	158.21	[7]
AgNPs-St-PEG-PAA	300	182.53	[7]
P3HT-CNT/Ti	200	164	[20]
PAA-MWCNTs	18	5.64	[22]
PNMA-AA/St	27.5	7.9	[24]
PAN-PRGO	150	164.79	[34]
P(AA-co-MA-co-DMTU)	400	198.23	[37]
PAA-DMDAAC/St	4000	191.97	[38]
CTS-g-PAA	3669	798.92	[40]
CTS-g-PAA/50%APT	3669	503.21	[41]
CJ-g-PAA	500	135	[42]
Cys-C@Fe_3_O_4_	100	94.33	[19]
MBI-OHTC	25	11.63	[27]
MBI-OHTC	50	21.52	[27]
PAA-AM/St	15	19.23	This work

Abbreviations in Table 3: AN—acrylonitrile; AMP—acrylonitrile; PEG—polyethylene glycol; AgNPs—silver nanoparticles; P3HT-3-Hexylthiophene; CNT—carbon nanotube; MWCNTs—multiwall carbon nanotubes; NMA—N-methylacrylamide; PRGO—partial reduction graphene oxide; MA—partial reduction graphene oxide; DMTU—dimethylthiourea; DMDAAC—dimethyl diallyl ammonium chloride; CTS—chitosan; APT—attapulgite; CJ—cassia javanica seed gum; Cys—cysteine; C—carbon; MBI-2-Mercaptobenzimidazole; OHTC—organophilic calcined hydrotalcite.

## Data Availability

The data presented in this study are available on request from the corresponding author.
